# Better forbearance, lower depression: Evidence based on heart rate variability

**DOI:** 10.3389/fpsyg.2022.1019402

**Published:** 2023-01-10

**Authors:** Tiehong Su, Xinwen Guo, Manhua Liu, Rong Xiao, Zhongju Xiao

**Affiliations:** ^1^Department of Psychology, School of Public Health, Southern Medical University, Guangzhou, China; ^2^Department of Physiology, School of Basic Medical Sciences, Key Laboratory of Psychiatric Disorders of Guangdong Province, Guangdong-Hong Kong-Macao Greater Bay Area Center for Brain Science and Brain-Inspired Intelligence, Key Laboratory of Mental Health of the Ministry of Education, Southern Medical University, Guangzhou, China; ^3^General Practice Center, The Seventh Affiliated Hospital, Southern Medical University, Foshan, China

**Keywords:** forbearance, depression, positive psychological resource, heart rate variability, acute stress tasks

## Abstract

**Background:**

The relationship between forbearance, a psychological resource, and depression has to date remained inconclusive. The present study investigated heart rate variability (HRV) reactivity to acute stressor tasks in participants with different levels of forbearance to discover how forbearance influences depressive emotions when facing adversity.

**Method:**

The study examined the relationship between forbearance and depression, comparing HRV reactivity to stressor tasks in participants with different levels of forbearance. The levels of reported forbearance were assessed by the Forbearance Scale (FS). The Patient Health Questionnaire-9 (PHQ-9) was used to assessed depression severity. HRV reactivity was evaluated at five stages: baseline, the active stressor task, the period of recovery after the active stressor task, the passive stressor task, the period of recovery after the passive stressor task.

**Results:**

FS scores had a significant negative correlation with PHQ-9 and a significant positive correlation with HRV; significant differences existed between the basal HRV in the higher and lower FS groups. In the passive stressor task and the period of recovery after the active stressor task, significantly different HRV responses were identified between the two groups.

**Discussion:**

Forbearance was correlated with depression and HRV. The present research found differences in HRV among subjects with different levels of forbearance in the baseline as well as stressor and recovery periods, suggesting that self-regulation dysfunction may exist among persons with lower levels of forbearance. Because of the higher levels of forbearance, the negative emotions of individuals caused by adversity are mitigated.

## 1. Introduction

Forbearance, which in Chinese culture refers to people regulating and controlling their emotions, attitudes, and behaviors, has received attention as a psychological resource. It is defined as a psychological quality whereby an individual can withstand desires, difficulties, and pressures; endure temporary unhappiness or humiliation in order to achieve their long-term interests or goals; handle conflict peacefully as well as being self-effacing; and regulate their emotions, attitudes, and behaviors (Yu and Xiao, 2020). It is also a virtue that can be defined in terms of the qualities of kindness and tolerance in Chinese philosophy (Han and Zhang, 2018). As a psychological resource, the levels of forbearance influence people’s level of depression (Deng et al., 2019; Yu and Xiao, 2020; Deng and Xiao, 2022), and is a means of maintaining social and personal relationships in the face of provocation (Ho and Liang, 2021). Long-term forbearance is important for protect the general health and reduce the depression’s onset tendency, whereas short-term forbearance is more beneficial for happiness (Aghababaei and Tabik, 2015). However, there are some negative interpretations of forbearance in Chinese culture; Chinese idioms such as “*To swallow an insult and humiliation silently*” and “*Take something (taunt, snub and so on) lying down*” indicate that forbearance also imply that individuals have to suppress their feelings of discomfort and control their behavior even when they do not want to do so. For instance, people have to tolerate the provocation of others when they themselves are defenseless or wronged because of the desire to accommodate others. These negative aspects suggest that forbearance perhaps predispose individuals to depression in certain environments, and some researchers consider forbearance as a negative coping strategy that increases the risk of depression and anxiety (Coiro et al., 2017). The conflicting findings of past research reveal the complexity of the psychology of forbearance. Nonetheless, the previous studies have concluded that forbearance is, to some extent, a positive psychological resource (Schnitker, 2012; Zahra Marhemati, 2017). Positive psychological resources refer to resources that individuals can use to positively cope with and respond to stress and adversity, and are increasingly recognized as associated with relief of depression (Celano et al., 2020; Shi et al., 2020). Self-regulation is an important positive psychological resource (Krkovic et al., 2018). Positive self-regulation enables individuals to better cope with the effects of negative emotions in the face of adversity (Ottenstein, 2020). Thus, further research was required to examine the relationship between forbearance and self-regulation in the context of a stressor, explaining the effect of forbearance on depressive symptoms. This would determine defining whether forbearance is a positive psychological resource and coping strategy.

The Psychophysiological Coherence Model theorizes that rhythmic activity in living systems can reflect the regulation of interconnected psychological, biological, and environmental networks (McCraty et al., 2009). Coherent and harmonious rhythms signify a stable and orderly living system associated with personal overall well-being, cognitive ability, socialization, and physical performance (McCraty and Childre, 2010). Heart rate variability (HRV) reflects one’s heart rhythm, and is an important non-invasive indicator often used to investigate cardiac autonomic nervous system (ANS) activity (Thayer et al., 2012). Examining HRV can be used to assess a person’s self-regulation ability in the face of stressors and is an indicator of psychological resilience and psychological flexibility (Visted et al., 2017; Lau et al., 2021). HRV analysis methods mainly focus on the time and frequency domains (TaskForce, 1996). Poor HRV is considered to be an indicator of many mental health problems (Kemp et al., 2014; Jiang et al., 2015; Guo et al., 2021); better emotional regulation, a lower perceived stress, and lower cognitive load are reflected by high HRV (Lischke et al., 2018; Dias et al., 2019).

The time and frequency domains are used to reflect the activity of the two branches of the ANS. Root mean square of R-R intervals (RMSSD) is one of the primary time-domain measures used to reflect the vagally mediated changes that occur in response to HRV. A lower RMSSD means that individuals are more likely to use maladaptive coping strategies in the face of stress (Machado et al., 2021). High-frequency power band (HF) can be used to evaluate the impact of parasympathetic nerves on the heart, and is highly correlated with respiratory sinus arrhythmia (RSA). RSA is a reliable biomarker of emotion regulation capacity in individuals (Beauchaine, 2015), which is thought to isolate the parasympathetic influence on the heart (Scott et al., 2021). A number of studies have confirmed that HF is an effective frequency domain measure of parasympathetic nerve activation (Balzarotti et al., 2017; Laborde et al., 2017; Kirk and Axelsen, 2020). Studies have found a negative relationship between HF and perceived emotional stress; reduced HF levels also reflect poor emotional inhibition (Gillie et al., 2014). Low-frequency power band (LF) with greater sympathetic sensitivity reflects baroreceptor activity (Shaffer et al., 2014; Pham et al., 2021), as well as the mixture of sympathetic and parasympathetic nerve contribution (Kidwell and Ellenbroek, 2018). Higher LF scores signify serious depression, anxiety, and perceived stress (Michels et al., 2013; Druzhkova et al., 2019). LF/HF is the ratio of LF to HF power; a high LF/HF ratio reflects greater sympathetic activity relative to parasympathetic activity (Thayer et al., 2012).

Acute laboratory stressors are used to elicit an immediate individuals’ stress response immediately from individuals. They can be classified according to the different responses from the parasympathetic and sympathetic nerves. One broad category of acute stressors requires the participant to generate a behavioral response (e.g., solving math problems quickly, generating an impromptu speech, or receiving a cold-pressor test). It has been confirmed that these types of stressors invoke parasympathetic withdrawal, sympathetic activation, as well as an increase in heart rate (Jiang et al., 2017; Renna et al., 2022); furthermore, they contribute to a rejection of environmental stimuli and make the participant generate a defensive or escape response. These kinds of acute stressors have been labeled as “active” tasks and “sensory rejection” tasks by researchers (Lacey and Lacey, 1958). The other broad category of acute stressors requires the participant to be presented with visual, auditory, or tactile stimuli (e.g., watching videos or images featuring blood and injury). These types of acute stressors will invoke parasympathetic activation and a decreased heart rate, promoting enhanced cognitive processing of the threat stimuli by taking in the environmental stimuli (Watford et al., 2020). Researchers have termed these “passive” and “sensory intake” tasks (Lacey and Lacey, 1958). Either type of acute stressors can elicit HRV reactivity. Rapid arithmetic or impromptu speech tasks in active stressors have been found to significantly reduce HF in HRV (Whited et al., 2014; Petrowski et al., 2017). In contrast, participants’ HF response was found to significantly increase when they were asked to complete passive tasks such as watching a violent or bloody video compared to an emotionally neutral video (Shenhav and Mendes, 2014).

In this study, we aimed to discover the effects of forbearance on depression from an ANS perspective. The primary purpose of our study was to compare the HRV reactivity of participants with different levels of forbearance in acute stressors and hence investigate whether forbearance as a psychological resource can influence the emotional regulation and stress-coping ability of individuals in distress.

## 2. Materials and methods

### 2.1. Participants

Participants from Southern Medical University were recruited through flyers posted on campus and a website link providing recruitment information. The study was conducted from March 2021 to January 2022, and we received responses from 161 potential participants, who were all screened according to the following inclusion criteria: (1) no history of heart disease and mental illness, such as arrhythmia, coronary heart disease, diagnosed depression, anxiety, insomnia, and so on; (2) no history of medication, including cardiovascular medications, psychotropic medications, and the contraceptive pill; (3) no alcohol or tobacco addiction. A total of 130 participants were recruited based on the initial screening results.

As shown in Table 1, the mean age of the participants was 22.03 (*SD* = 2.04), while the average BMI was 20.40 (*SD* = 2.39). Participants reported moderate levels of caffeine consumption and engagement in sport. This study was approved by the Ethics Committee of the School of Public Health, Southern Medical University, and informed consent was given by all participants.

**Table 1:** Descriptive statistics of demographic data from higher and lower FS groups.

	Higher FS (*n* = 66)	Lower FS (*n* = 64)	Value of *p*
Age, year (mean ± *SD*)	21.94 ± 2.13	22.13 ± 1.95	0.606
BMI (mean ± *SD*)	20.64 ± 2.47	20.16 ± 2.30	0.262
Gender			0.491
Female (*n*, %)	48 (72.73%)	43 (67.19%)	
Exercise frequency per week (*n*, %)			0.380
0	16 (24.24%)	18 (28.13%)	
1–3	38 (57.58%)	29 (45.31%)	
3–5	8 (12.12%)	14 (21.88%)	
>5	4 (6.06%)	3 (4.68%)	
Caffeine consumption frequency per week (*n*, %)			0.371
Never	52 (78.78%)	47 (73.44%)	
Once per week	7 (10.61%)	5 (7.81%)	
A few times a week	5 (7.58%)	11 (17.19%)	
Once a day or more	2 (3.03%)	1 (1.56%)	
Total PHQ-9 score	6.29 ± 4.36	7.52 ± 3.60	0.083
Total FS score	80.89 ± 5.04	67.63 ± 5.05	**<0.001**

*p*-values in bold indicate significant difference (*p* < 0.05) between the two groups. BMI, Body Mass Index; PHQ-9, Patient Health Questionnaire-9; FS, Forbearance Scale.

### 2.2. Study procedure

Participants arrived at the lab to complete the informed consent form and questionnaires. Following this, an ECG (electrocardiograph) electrode was placed on each individual’s sternum. After placement, participants were required to sit quietly for 15 min while the baseline ECG data were recorded. A stress induction was then given; participants were asked to complete the first stress task (active stressor, 6 min) and then to sit quietly for recovery (6 min). The second stressor was then administered (passive stressor, 6 min) followed by a recovery condition (6 min). Finally, participants were instructed to keep relaxed and sit quietly for 15 min while the post-ECG data were recorded. The order of stressor presentation shown to each participant was randomized. Throughout the experimental period, ECG data were recorded.

### 2.3. Study design

#### 2.3.1. Baseline condition

Participants were instructed to avoid caffeine, alcohol, and exercise for 24 h before the experiment. They arrived at the lab at the appointed time (8:30 a.m. or 10:30 a.m.). After self-reported psychological measures were completed and the electrode was placed, each participant was asked to sit quietly for 15 min, while the baseline ECG data were recorded; the data were collected under a controlled temperature (24–26°C).

#### 2.3.2. Active stressor

In this study, the math component of the Trier Social Stress Task (TSST) was used as an active stressor; it has been confirmed to significantly invoke an acute stress response in individuals, including an increase of salivary cortisol (Jiang et al., 2017), heart rate (Woody et al., 2017) and HRV responses (Petrowski et al., 2017). All participants received the active stressor. In this stressor, participants were required to start at the number 1,022 and quickly subtract 13 consecutively in front of a committee (made up of one man and one woman); they were asked to restart this task from the beginning if they made any errors. The stressor duration was 6 min.

#### 2.3.3. Passive stressor

An emotionally stressful video was used as the passive stressor. Videos containing violence, blood, or injury typically invoke parasympathetic activation and elicit a stress state (Brzozowski et al., 2018). Compared with a horror movie clip or a video of a car accident, a surgery video not only intuitively presents factors like trauma and blood, but also avoids other anxiety-inducing stimuli (Watford et al., 2020). Hence, a video of open-heart surgery was chosen to expose participants to blood and injury. The participants were randomly divided into a stress group and a control group according to a 3:1 ratio in order to ensure that this video did invoke acute stress in them (Aversano et al., 2012; Moher et al., 2012; Chandereng et al., 2020). Following randomization, subjects in the stress group were shown an edited video of a thoracotomy, while subjects in the control group were presented with an emotionally neutral clip about scenery. Both videos were played without sound to avoid the effect of background music on the experimental results. The stressor duration was 6 min.

#### 2.3.4. Recovery condition

Participants were instructed to sit quietly for 6 min after each stressor condition ended. During this time, the ECG data were continuously recorded.

### 2.4. Measures

#### 2.4.1. Heart rate variability

ECG data were recorded using a Holter monitor (Mobio^®^ Portable Recorder, Chengdu Synwing Technology Co., Ltd., Chengdu, China). ECG analytics 2.0.2 software was used to analyze the recorded data and obtain time and frequency domain indices. Using ECG analytics 2.0.2 software, ECG signals were visually inspected and corrected for artifacts or signal noise. The time-domain index of HRV is RMSSD, which is one of the primary time-domain measures (Kidwell and Ellenbroek, 2018), and one of the more commonly used time-domain indices in short-term recording (Shaffer and Ginsberg, 2017; Saboul and Hautier, 2019). Frequency domain indices of HRV include HF, LF, and LF/HF ratio. HF is mainly regulated by parasympathetic activity (Shaffer et al., 2014), while LF is modulated by both sympathetic and parasympathetic activity (Xhyheri et al., 2012). The LF/HF ratio was computed as an index to reflect the sympathovagal balance (Pham et al., 2021). Absolute power was generated for HF and LF; the indices used in the current research were HF (n.u.) and LF (n.u.), which can be obtained accurately in a short-term measure (TaskForce, 1996; Shaffer and Ginsberg, 2017).

#### 2.4.2. Forbearance scale

Levels of forbearance were assessed using the Forbearance Scale (FS; Yu and Xiao, 2020), which is based on the understanding of the word “*Ren-Nai* (Forbearance)” in Chinese traditional culture. It aims to measure the ability of individuals to adjust their emotions, attitudes, and behaviors in life events in accordance with Chinese cultural characteristics. The FS contains five factors: repressive avoidance, restraint and concession, patience and peace, delayed gratification, and positive cognition. It can reflect the forbearance characteristics of an individual in four aspects: cognition (positive cognition), behavior (repressive avoidance, restraint, and concession), motivation (delayed gratification), and personality (patience and peace). In the present study, the internal consistency of the FS was good (*α* = 0.866). In a previous study, application of the FS to different social groups showed that FS scores were significantly negatively correlated with depression (Yu and Xiao, 2020; Deng and Xiao, 2022). The FS consists of 20 items, which are scored on a scale from 1 (totally inconsistent) to 5 (totally consistent). The total score on the scale is 20–100; higher total scores indicate a higher level of forbearance.

#### 2.4.3. Patient health Questionnaire-9

Depression severity was assessed by the Patient Health Questionnaire-9 (PHQ-9). The PHQ-9 consists of nine items, scored on a scale from 0 (not at all) to 3 (nearly every day); the maximum score is 27. Higher total scores mean higher levels of depressive symptoms: a score of 0–4 indicates minimal depression, 5–9 indicates mild depression, 10–14 moderate depression, 15–19 moderately severe depression, and 20–27 serious depression. The Chinese version has good reliability and validity. The Cronbach’s alpha score for the scale in this study was 0.844.

### 2.5. Statistical analysis

All participants were divided into higher FS and lower FS groups based on the mean FS scores. Demographic data were analyzed by descriptive analysis. The Kolmogorov–Smirnov test was applied to all study variables to determine whether the data were normally distributed, while a Spearman’s rank correlation analysis was used to evaluate the relationship between FS and PHQ-9 scores. The relationship between FS scores and baseline HRV was also tested by Spearman’s rank correlation analysis. A non-parametric test (Mann–Whitney *U*-test) and Student’s *t*-test was used to compare the differences in HRV responses between the higher FS and lower FS groups at baseline, under different stressors, recovery conditions, and post-stressor period. All data calculations were performed using SPSS 26.0. A *p*-value < 0.05 was used to confirm statistical significance.

## 3. Results

### 3.1. Demographic data

The demographic data are presented in Table 1. The higher FS group and lower FS group did not differ in terms of age, BMI, gender ratio, exercise frequency per week, and caffeine consumption frequency per week.

### 3.2. Correlations between FS, depression, and heart rate variability at baseline

Correlations were analyzed between the FS items and PHQ-9 in order to verify the relationship between forbearance and depression, as shown in Table 2. The results showed a significant negative correlation between the total FS score and depressive symptoms (*r* = −0.233, *p* = 0.008). In addition, the results of the correlation analysis between individual FS items and depressive symptoms showed that higher levels of self-reported patience and calmness, delay of gratification, and positive identification with the concept of forbearance (items 3, 7, 8, 12, 13, 18, and 20) were significantly and negatively associated with depressive symptoms. In contrast, items related to suppressing negative emotions and evasive behavior in distress (items 1, 6) were significantly positively correlated with depression.

**Table 2:** Correlations between FS and PHQ-9.

FS item	*r*
1. A lot of times I wronged myself because of accommodating others	0.171*
2. I can tolerate the current unhappiness in order to the longer-term goal	−0.120
3. I am a patient person	−0.309**
4. I’d rather be compromised than haggle over every ounce with others	0.021
5. I think moderate be compromised is more conducive to success	−0.140
6. You must learn to swallow hard when your ability is insufficient	0.192*
7. I do not care about the immediate gains and losses, but about the future development	−0.290**
8. I can control my negative emotions very well	−0.350**
9. I try to avoid conflict with others	−0.088
10. I believe that people have to bear hardships and withstand hard work to have better development	−0.120
11. Sometimes forced by the situation, people should learn to compromise	−0.020
12. I am willing to restrain my current desires and needs for future development	−0.322**
13. I can control of my speech and behavior when I am upset or angry	−0.379**
14. I think I would rather make concessions to avoid trouble than tit for tat	−0.125
15. Forbearance is the embodiment of personal cultivation	−0.115
16. when we cannot get out of predicament, we should learn to accepting the adversity	0.098
17. I am willing to sacrifice my immediate interests for future gains	−0.055
18. I rarely vent my bad emotions to others	−0.173*
19. I rarely argue with others to get the Short-time upper hand	−0.122
20. People who are good at forbearance will have more opportunities for development	−0.279**
FS total	−0.233**

Items were reverse scored so that: 1 = Totally inconsistent, 2 = Partly inconsistent, 3 = Not sure, 4 = Partly consistent, 5 = Totally consistent. Thus, higher scores indicate higher levels of reported forbearance.

*p < 0.05, **p < 0.01.

Correlation analysis was conducted to assess the degree of interdependence between FS and HRV. The results were as follows: RMSSD, *r* = 0.301, *p* < 0.001; HF, *r* = 0.220, *p* = 0.012; LF, *r* = −0.219, *p* = 0.012; LF/HF, *r* = −0.190, *p* = 0.30. These results indicate a relationship between FS and HRV.

### 3.3. Forbearance and heart rate variability at baseline

Table 3 shows data related to the baseline HRV indices for the higher FS and lower FS groups; the results show a significant difference in the baseline HRV in the two groups.

**Table 3:** Between-group comparisons at baseline HRV in higher FS group and lower FS group.

Index	Higher FS (*n* = 66)	Lower FS (*n* = 64)	Value of *p*
Baseline-RMSSD (ms)	36.30 (25.5–50.13)	27.00 (20.78–38.13)	**0.007** ^b^
Baseline-HF (n.u.)	48.28 ± 16.79	41.98 ± 17.18	**0.036** ^a^
Baseline-LF (n.u.)	47.02 ± 16.63	53.88 ± 17.39	**0.023** ^a^
Baseline-LF/HF	0.99 (0.59–1.56)	1.31 (0.80–2.61)	**0.031** ^b^
Baseline-HR, beat/min	74.85 ± 9.68	78.09 ± 9.37	0.061^a^

Mean values (±SE) for the indices of HF, LF and HR; Median values (Q1–Q3) for the indices of RMSSD and LF/HF. *p*-values in bold indicate significant difference (*p* < 0.05) between the two groups.

a Student’s *t*-test.

b Mann–Whitney *U*-test.

### 3.4. Forbearance and heart rate variability reactivity in stressor stages

#### 3.4.1. Heart rate variability reactivity in passive stressor in stress and control groups

The Wilcoxon signed-rank test was used to investigate the difference between HRV at baseline and HRV at the passive stressor stage in the stress and control groups. Regarding HRV reactivity in the stress group, significant differences were found between the baseline and passive stressor stages (Table 4). In the control group, the results showed no significant differences between the two stages (Table 4). Hence, we can be certain that the thoracotomy video did cause stress in the subjects. Additionally, an interaction effect of 2 (higher/lower FS) × 2 (stress/control) was not significant.

**Table 4:** HRV indices before and after the passive stressor in the stress and control groups.

Index	Emotionally stressed group (*n* = 100)		Value of *p*	Controls (*n* = 30)		Value of *p*
	Baseline	Passive stress		Baseline	Passive stress	
RMSSD (ms)	32.50 (20.78–41.30)	38.05 (26.83–50.98) *	<0.001	35.80 (22.53–48.23)	34.25 (23.20–49.35)	0.376
HF (n.u.)	45.50 (33.63–59.60)	52.15 (39.70–67.83) *	<0.001	47.05 (33.43–53.20)	40.85 (29.75–55.38)	0.614
LF (n.u.)	49.75 (35.55–63.38)	42.55 (27.58–56.98) *	<0.001	47.50 (41.05–59.43)	52.45 (39.53–66.60)	0.517
LF/HF	1.09 (0.61–1.85)	0.81 (0.41–1.51)*	<0.001	0.99 (0.76–1.59)	1.39 (0.69–2.23)	0.504
HR, beats/min	76.00 (70.25–83.75)	73.00 (68.25–79.00) *	<0.001	72.50 (66.75–85.00)	72.50 (64.00–83.00)	0.205

Median values (Q1–Q3) for the indices of each group.

*p < 0.05 vs baseline.

#### 3.4.2. Heart rate variability reactivity in stressor and recovery stages in the two groups

Significant differences were found in the passive stress task. The RMSSD and HF for the higher FS group were significantly higher than in the lower group, while the reverse results were found for the LF and LF/HF ratio (Table 5). Interestingly, regarding the HRV reactivity in the active stressor, no significant differences between the two groups were observed for all indices (all *p*-values > 0.05). However, regarding HRV reactivity in the higher FS and lower FS groups, there was a significant difference in the recovery period after the active stress task (Table 5).

**Table 5:** HRV indices between the recovery stage after the active stressor and the passive stressor in the higher and lower FS groups.

Index	Recovery stage after active stressor		Value of *p*	Passive stressor stage		Value of *p*
	Higher FS (*n* = 66)	Lower FS (*n* = 64)		Higher FS (*n* = 49)	Lower FS (*n* = 51)	
RMSSD (ms)	36.80 (28.20–48.75)	29.40 (23.80–37.80)	**0.005** ^b^	46.93 ± 25.25	36.63 ± 15.98	**0.006** ^a^
HF (n.u.)	27.40 (17.30–39.20)	22.90 (15.30–30.50)	**0.029** ^b^	60.10 (44.75–73.70)	47.20 (34.50–65.60)	**0.010** ^b^
LF (n.u.)	67.70 (53.35–79.15)	73.70 (67.10–81.70)	**0.050** ^b^	41.31 ± 18.72	49.49 ± 18.70	**0.014** ^a^
LF/HF	2.52 (1.36–4.56)	3.32 (2.22–5.16)	**0.028** ^b^	0.59 (0.32–1.19)	0.98 (0.43–1.81)	**0.027** ^b^
HR, beats/min	78.00 (72.00–84.50)	79.00 (74.00–86.00)	0.343^b^	72.00 (67.00–78.50)	74.00 (69.00–79.00)	0.274^b^

Mean values (±SE) for the indices of stressor stage RMSSD and LF; Median values (Q1–Q3) for the other indices. *p*-values in bold indicate significant difference (*p* < 0.05) between the two groups.

a Student’s *t*-test.

b Mann–Whitney *U*-test.

### 3.5. RMSSD reactivity during the whole procedure

**Figure 1:**
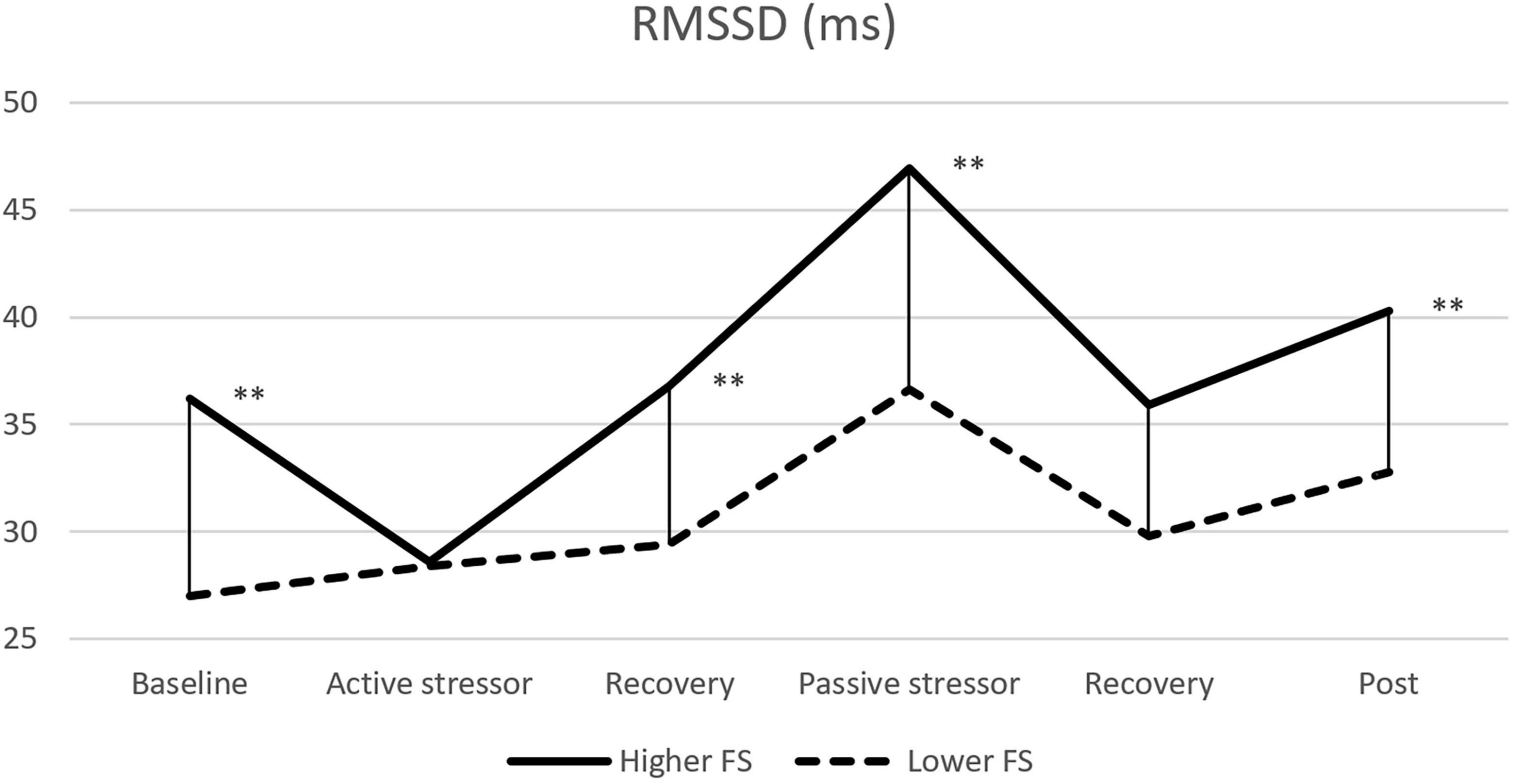
Comparisons of RMSSD reactivity in all stages between the higher and lower FS groups. **p < 0.01.

As the primary time-domain measure, the RMSSD is used to evaluate vagally mediated changes (Kidwell and Ellenbroek, 2018). The RMSSD of the higher and lower FS groups were compared at all stages (baseline, stressor, recovery, and post) using the Student’s *t-*test or Mann–Whitney *U*-test. A significant difference in RMSSD was found between the two groups in both the baseline and post periods. As shown in Figure 1, in the active stressor stage, there was a non-significant difference in RMSSD between the two groups because of a rapid decrease in RMSSD in the higher FS group during this time (all *p*-values > 0.05). However, there was a significant RMSSD rebound in the subsequent recovery stage in the higher FS group, resulting in a significant difference again with the lower group. In the passive stressor stage, the RMSSD in the higher FS group was significantly higher than that in the lower FS group, and there was no significant difference in the subsequent recovery stage (all *p*-values > 0.05).

## 4. Discussion

The main findings of this study are as follows: (1) there was a significant negative correlation between the levels of forbearance and depressive symptoms; (2) there was a significant positive correlation between the levels of forbearance and HRV; (3) there were significant differences in HRV between subjects with higher and lower FS at baseline, in the passive stress task, and in the recovery stage after the active stressor.

The relationship between FS scores and PHQ-9 scores was found to be statistically significant. Specifically, individuals with higher FS scores had fewer depressive symptoms than those with lower FS scores. Additionally, the correlation analysis between each FS item and the total PHQ-9 scores showed that the positive components of forbearance, such as personal traits of being patient and peaceful in daily life, delaying gratification in order to achieve better goals, and positive identification with the concept of forbearance, were negatively related to depression severity. In contrast, negative components such as avoidance behavior in the face of difficult situations or suppressing painful emotions were positively related to depression. This finding shows that as a psychological resource, forbearance has both positive and negative aspects. A recent positive psychology emphasized a new approach to the study of life and meaning through a subtle appreciation of the negative and positive sides of situations (Mayer and Vanderheiden, 2020). Forbearance has this exact same quality. Its positive side emphasizes the individual’s initiative to regulate their emotions, attitudes, and behaviors for the long-term good, while its negative side emphasizes its role in our positive functioning and transformation (Ivtzan et al., 2015).

Participants with higher FS scores had a higher RMSSD and HF, and lower LF and LF/HF ratio. Specifically, higher FS scores mean greater parasympathetic activity, while lower FS scores mean increased sympathetic activation. According to a neurovisceral integration model, a higher resting HRV is associated with more flexible emotional response and better use of adaptive regulatory strategies (Balzarotti et al., 2017). The positive emotions brought about by the mentality of forbearance promote an orderly cardiac rhythm, which then increases parasympathetic activity (representing an increase in HRV), and this increased activity in turn enhances a person’s positive emotions and experiences of well-being (McCraty et al., 2009). In contrast, lower levels of forbearance may create higher levels of depression, anger, anxiety, and worry. These negative feelings lead to cardiac rhythm disorder and ANS desynchronization, leading to a further decline in HRV and even affecting the activity of the prefrontal cortex, resulting in more emotional and cognitive dissonance (McCraty et al., 1995).

In the passive stressor (i.e., watching an emotionally stressful video), forbearance demonstrated an effect on mental health. Individuals with higher FS scores showed better HRV reactivity, as demonstrated by higher RMSSD and HF and lower LF and LF/HF. Although watching negative emotional films or images causes an increase in those indices (Shenhav and Mendes, 2014; Brzozowski et al., 2018), and the lower FS group also presented an upward trend, it is clear that subjects with higher FS scores had a larger increase in HF power. HF has been confirmed as being related to greater inhibitory control and more successful suppression of negative emotions; a person with higher HF is therefore less exposed to emotional stress (Gillie et al., 2014; Beauchaine, 2015). Additionally, the higher and lower FS groups presented the same trend of a decrease in LF power in the passive stressor. However, the lower FS group had a higher LF response, which may indicate that participants in the lower FS group felt more stress when viewing the stressful video. A higher LF response might imply parasympathetic blunting in individuals with low forbearance. In the face of a stressor, the baroreceptors generate action potentials that lead to sympathetic inhibition and parasympathetic activation *via* the medulla, thereby promoting the body’s balance and recovery (Shaffer et al., 2014). However, the blunted parasympathetic nerves of a person with low forbearance are difficult to activate in time when receiving signals from the baroreceptors, resulting in an imbalance of the ANS, which makes it difficult to effectively regulate and adapt to emotional stress caused by adversity. The LF/HF ratio also presented the same reactivity; our findings showed that the LF/HF of lower FS individuals was higher than people with high FS scores in the passive stress task and this difference was shown throughout the experiment. A higher LF/HF indicates that sympathetic activity is higher than parasympathetic activity (Shaffer et al., 2014). These results confirm our previous interpretation: parasympathetic nerves are blunted, while sympathetic ones are dominant in the ANS of persons with low forbearance.

There was no significant difference in HRV reactivity between the higher and lower FS groups in the active stress task (i.e., solving math problems). As also found in most previous studies, all participants experienced a decrease in HF, increase in LF, and a rapid acceleration of heart rate while completing the active stress task. This suggests that the active stressor in the study is effective for stimulating sympathetic activation and parasympathetic withdrawal (Watford et al., 2020; Renna et al., 2022). Interestingly, the RMSSD of the higher FS group presented a substantial decrease under this stage, so that it was close to the value of the lower FS group; see Figure 1. Researchers have found that individuals who tend to use maladaptive coping strategies show a greater reduction in RMSSD during speech tasks, while people with good adaptive strategies maintain the magnitude of RMSSD when undergoing this stress (Machado et al., 2021). However, the current study found that participants with higher FS scores showed a more substantial decrease in RMSSD, whereas those with lower FS scores maintained it. As mentioned above, forbearance has both positive and negative connotations. People with high levels of forbearance tend to adopt an inward psychological defense mechanism when they are under the unfavorable evaluation of others or in a pressured environment, but people with lower levels of forbearance refuse this kind of withdrawing behavior and are more willing to use their own resistance to face these conditions. Participants were required to complete tasks such as solving math problems or making an impromptu speech when they are evaluated by others in the active stressor. Therefore, subjects with higher FS scores had a rapid decline in RMSSD in such stress tasks. This finding further clarifies some of the negative characteristics of forbearance, but raises the question of why did most previous studies suggest that forbearance is beneficial to an individual’s mental health? Indeed, we found that although the higher FS group experienced a decrease in HRV during the stressor, a rapid compensation of HRV occurred in the subsequent recovery period, which again formed a significant difference with the lower FS group; see Figure 1. In short, the higher FS group had better HRV recovery performance after experiencing the active stressor. Let us associate this finding with the real-life context: people with higher levels of forbearance endure unhappiness and then move forward with high morale; people with lower levels of forbearance achieve staged victories through catharsis and resistance under stress, but cannot get rid of the negative effects of stressor. Interestingly, previous studies reported that higher HRV in the recovery stage is linked to behaviors of actively seeking social support (Geisler et al., 2013) and greater resilience to stress (An et al., 2020), while lower HRV in the rest stage is associated with anxiety, depression, and increased risk of cardiovascular disease (Chalmers et al., 2014). McCraty and Atkinson pointed out that individuals who are good at self-regulating their emotions have a faster recovery rate after experiencing stress (McCraty and Atkinson, 2012). Hence, although people with higher levels of forbearance use inappropriate behavioral responses when faced with active stressors, parasympathetic dominance after experiencing stress will help them to better recover from the stressor and reduce its negative effects.

## 5. Limitations of the study

Several limitations of this study must also be mentioned. First, the age range of the participants was between 18 and 29 years old; our study lacked exploration of adolescents, middle-aged, and elderly groups. Further studies should expand the age range of subjects to improve the external validity of the conclusions. Second, the current study did not measure participants’ subjective reporting of stressfulness after each stress task. In future investigations, self-report data on stressfulness need to be collected. Third, the stress tasks used in our study were conducted under a condition of acute laboratory stress, which provides a good degree of control, but cannot completely cover all the stress experienced by individuals in reality. Chronic stressors are one of the sources that affect individuals’ mental health. Therefore, future research could explore whether forbearance has an effect on chronic stressors (e.g., academic stress, parenting stress, etc.).

## 6. Conclusion

Forbearance is associated with autonomic responses represented by HRV. The study found a negative correlation between FS scores and depression, and a positive correlation with HRV. Participants with high levels of forbearance had a higher resting HRV and better HRV reactivity in the face of stressors. Although participants with high levels of forbearance had inappropriate behavioral responses to one of these stressors, the subsequent HRV rebound remained protective of mental health. As a significant part of Chinese culture, forbearance has an important influence on the Chinese. Our study confirmed the positive significance of forbearance in psychology, and clarified the role of forbearance in emotion regulation and relief of depressive symptoms.

## Data availability statement

The raw data supporting the conclusions of this article will be made available by the authors, without undue reservation.

## Ethics statement

The studies involving human participants were reviewed and approved by the Ethics Committee of the School of Public Health, Southern Medical University. The patients/participants provided their written informed consent to participate in this study.

## Author contributions

TS, XG, and ML collected and analyzed the data. TS and RX interpreted the data and wrote the first draft of the manuscript. ZX and TS generated the idea, designed the study, and wrote the manuscript. All authors contributed to the article and approved the submitted version.

## Funding

This work was supported by grants from Key Laboratory of Psychiatric Disorders of Guangdong Province and National Natural Science Foundation of China to ZX (32070994 and 31872769).

## Conflict of interest

The authors declare that the research was conducted in the absence of any commercial or financial relationships that could be construed as a potential conflict of interest.

## Publisher’s note

All claims expressed in this article are solely those of the authors and do not necessarily represent those of their affiliated organizations, or those of the publisher, the editors and the reviewers. Any product that may be evaluated in this article, or claim that may be made by its manufacturer, is not guaranteed or endorsed by the publisher.
